# Techniques for the Assessment of In Vitro and In Vivo Antifungal Combinations

**DOI:** 10.3390/jof7020113

**Published:** 2021-02-04

**Authors:** Anne-Laure Bidaud, Patrick Schwarz, Guillaume Herbreteau, Eric Dannaoui

**Affiliations:** 1 Parasitology-Mycology Unit, Microbiology Department, APHP, European Georges Pompidou Hospital, Paris-Descartes University, F-75015 Paris, France; anne-laure.bidaud@aphp.fr; 2 Department of Internal Medicine, Respiratory and Critical Care Medicine, University Hospital Marburg, Baldingerstraße, D-35043 Marburg, Germany; patrick.schwarz@med.uni-marburg.de; 3 Center for Invasive Mycoses and Antifungals, Philipps University Marburg, D-35037 Marburg, Germany; 4 Department of Biochemistry, Nantes University Hospital, F-44093 Nantes, France; guillaume.herbreteau@chu-nantes.fr; 5 Dynamyc Research Group, Paris Est Créteil University (UPEC, EnvA), F-94010 Paris, France

**Keywords:** antifungal resistance, antifungal combination, checkerboard, time-kill curves, agar diffusion assay, gradient concentration strip

## Abstract

Systemic fungal infections are associated with high mortality rates despite adequate treatment. Moreover, acquired resistance to antifungals is increasing, which further complicates the therapeutic management. One strategy to overcome antifungal resistance is to use antifungal combinations. In vitro, several techniques are used to assess drug interactions, such as the broth microdilution checkerboard, agar-diffusion methods, and time-kill curves. Currently, the most widely used technique is the checkerboard method. The aim of all these techniques is to determine if the interaction between antifungal agents is synergistic, indifferent, or antagonistic. However, the interpretation of the results remains difficult. Several methods of analysis can be used, based on different theories. The most commonly used method is the calculation of the fractional inhibitory concentration index. Determination of the usefulness of combination treatments in patients needs well-conducted clinical trials, which are difficult. It is therefore important to study antifungal combinations in vivo, in experimental animal models of fungal infections. Although mammalian models have mostly been used, new alternative animal models in invertebrates look promising. To evaluate the antifungal efficacy, the most commonly used criteria are the mortality rate and the fungal load in the target organs.

## 1. Introduction

Fungal infections are serious pathologies that, despite adequate treatment, have high mortality rates [1,2]. In addition, besides natural resistance in some species, acquired resistance to antifungals is increasing [3,4]. Therefore, new therapeutic alternatives are needed. At present, only a few antifungals belonging to a limited number of antifungal classes with different mechanisms of action are on the market [3]. Despite the urgent need for new antifungals and antifungal classes [5], a promising therapeutic strategy would be to use antifungals in combination. Indeed, one of the main advantages of combining antifungals is to overcome resistance [6]. Moreover, antifungal combination can increase the efficacy of the combined molecules yielding to synergy. Combination therapy can also reduce toxicity by decreasing antifungal dosages, and improve the pharmacokinetics of one or both molecules [7]. Antifungal combinations are already used in clinical practice, such as 5-flucytosine combined with amphotericin B as first-line treatment for cryptococcal meningitis [8]. Moreover, it is also important to know if a combination exhibits antagonism.

**Figure 1:**
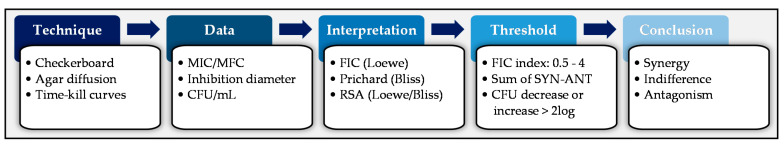
Summary of steps needed to perform and interpret antifungal combination tests. MIC, minimal inhibitory concentration; MFC, minimal fungicidal concentration; CFU, colony forming unit; FIC, fractional inhibitory concentration; RSA, response-surface analysis; SYN, synergy; ANT, antagonism.

Regarding the general approach of studying antifungal combinations, several steps are needed to perform and interpret antifungal combination tests (Figure 1). The first step is to choose an experimental technique: a liquid dilution method (e.g., checkerboard), a method of agar diffusion (e.g., gradient concentration strips such as Etest), or a study of fungicidal effect (e.g., time-kill). Using these methods, raw numerical data are obtained: minimal inhibitory concentrations (MIC), inhibition diameters, or number of colony-forming units (CFU) over time. The MIC data for example, are then analyzed, either using a graphical method (surface analysis), or by calculation of the inhibitory fractional concentration index (FIC index), and interpreted according to consensual thresholds or predetermined criteria. Finally, based on the results, a mode of interaction that is synergy, indifference (no interaction), or antagonism can be concluded. Currently, none of these steps are standardized, and therefore a large number of variables can influence the final results.

## 2. In Vitro Techniques

To study antifungal combinations, several experimental techniques are possible. Each method has advantages, but also disadvantages (Table 1)**.**

### 2.1. Liquid Microdilution Technique: Checkerboard

**Figure 2:**
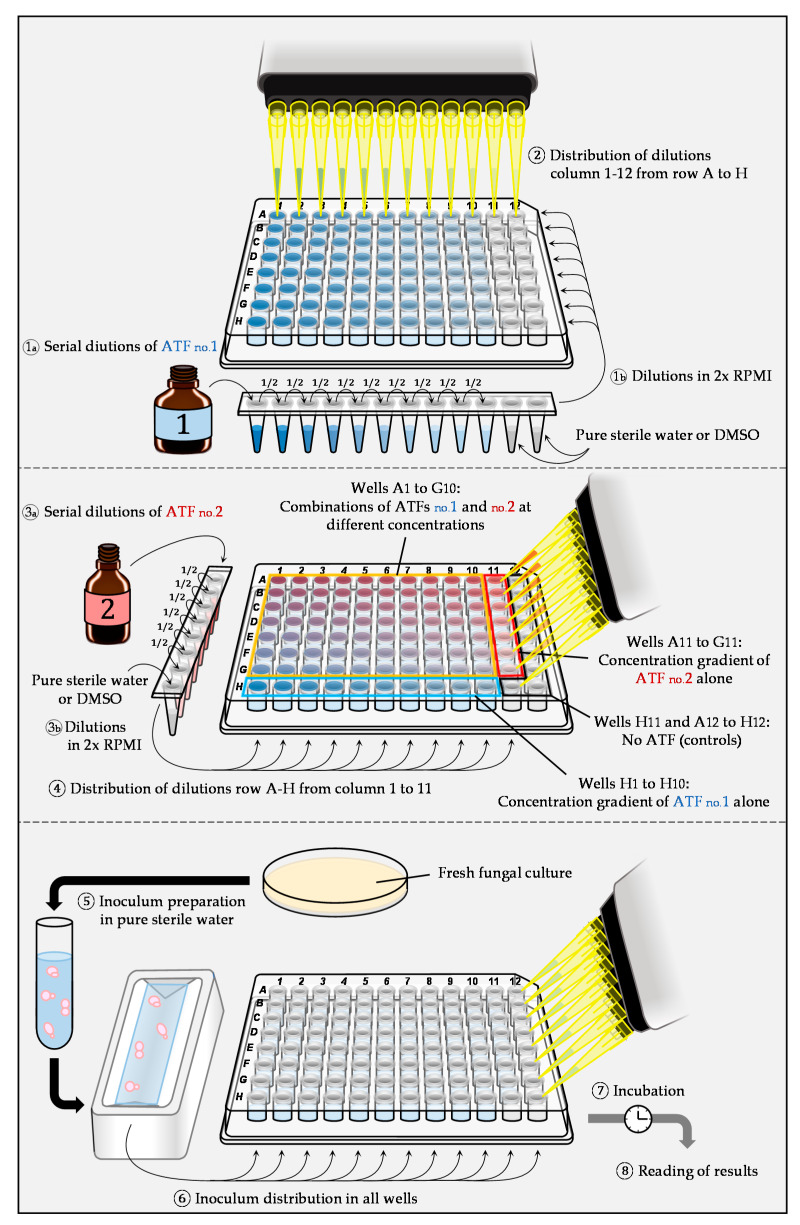
Example of preparation and inoculation of microplates using the checkerboard method based on the EUCAST methodology for antifungal susceptibility testing.

The checkerboard method is generally based on the standardized EUCAST [9] or CLSI [10] broth micro-dilution techniques and performed in 96-well microplates [7,11]. Initially, each drug is diluted in series, usually using a dilution factor of two. These solutions are added to the culture medium (Roswell Park Memorial Institute Medium, RPMI), which is then distributed in a 96-well microplate (Figure 2). After preparation of the microplates, each well is inoculated with the fungal inoculum (yeast cells or conidia), and microplates are then incubated. To be able to interpret the results correctly, sufficient two-fold dilutions below and above the MIC have to be included for each antifungal. Reading can be performed either visually or spectrophotometrically. Nevertheless, for a more objective MIC determination and possible automation, the spectrophotometric method should be preferred [7,12]. After reading of the microplates, the quantitative data can be analyzed in different ways.

The checkerboard is the most often used in vitro technique [11,13], and is therefore considered to be the “reference” method, even though there is currently no consensus regarding the reference technique to be used for assessing antifungal combinations. Nevertheless, this technique has some drawbacks, in particular, the range of concentrations tested is discontinuous and the dilutions are performed in a geometric manner. This means that only certain combinations of concentrations can be evaluated on the microplate, and the error in determining the MICs is not the same over the entire concentration range. The checkerboard technique can also be used to test triple combinations [14]. It has been used to test triple combinations in the field of antivirals (e.g., against HIV) [15], antibiotics (e.g., against Mycobacteriacae and Enterobacteriacae) [16,17], and antifungal agents against *Aspergillus* spp. [18], *Cryptococcus neoformans* [19,20], *Candida albicans* [21,22,23], Mucorales [24], and *Scedosporium* spp. [25,26].

### 2.2. Agar-Medium Diffusion Techniques

Agar-medium diffusion techniques are widely used to determine antifungal susceptibilities. These methods can be adapted in different ways to study antifungal combinations.

#### 2.2.1. Disk Diffusion Method

Disks impregnated with one of the two antifungal agents are placed face to face on an agar previously inoculated with the strain to be studied. The optimal distance between the discs to visualize the interaction should be determined in preliminary experiments. After growth of the microorganism, growth inhibition zones are obtained around each of the disks. In the zone were the diffusion of both antifungals is overlapping, special inhibition zones can be recognized. Depending on the growth characteristic of the strain on these zones, the interaction can be concluded.

Another technique is to use a disk impregnated with an antifungal agent, while the second antifungal is incorporated into the agar at a sub-inhibitory concentration. The inhibition zone obtained is compared to that of the control, i.e., agar without an antifungal agent. Compared to the control agar, an increase or decrease of the inhibition diameter around the disk will be obtained in cases of synergy or antagonism, respectively. Another way to detect antagonism is to incorporate the antifungal in the agar at a concentration higher than the MIC of the strain. In case of antagonism, growth of the microorganism will occur only around the disk [27].

This method has been used to evaluate antifungal combinations or combinations of antifungals with non-antifungal drugs against *Candida* spp. [28,29,30], *Cryptococcus* spp. [30], and dermatophytes [31].

#### 2.2.2. Right Angle Scattering Method

One of the common methods to assess interactions of antimicrobial drugs is the right angle scattering method [32]. It consists of placing two drug-impregnated paper strips at right angles on an agar plate. Depending on the growth characteristics of the microorganism in the area, where drug diffusion into the agar is overlapping, either synergy, indifference, or antagonism can be concluded. The technique is easy and fast to perform, but has only seldom been used for assessing antifungal combinations [33]. The fact that the technique is a diffusion method makes it possible to obtain a continuous gradient of the concentrations of the antifungals. However, the method has also several drawbacks. It is only qualitative, and the interpretation remains subjective as it depends on the growth of the microorganism on only a few millimeter-wide overlapping zone of the antifungals, which may vary between the experiments [7,27]. Additionally, the choice of concentrations of the antifungals on the paper strips makes preliminary experiments necessary.

#### 2.2.3. Gradient Concentration Strip (Etest) method

Gradient concentration strips allow researchers to measure the MICs of antifungals. Strips are impregnated with concentration gradients of the molecules [34]. Even though this is not the reference method for antifungal susceptibility testing, it is a simple test to determine MICs. Gradient concentration strips can also be used to test interactions between drugs [7,27,35]. The endpoints used for MIC determination (complete or partial inhibition) for antifungal combination are the same as those used when drugs are tested alone. Due to the existence of registered trademarks (e.g., Etest), the reproducibility of the technique is good. Several methods are used to assess antifungal combinations.

The first method is used when strips are commercially available for both antifungals. After determination of the MICs alone, the MIC in combination can be evaluated in three different ways.

(i) The cross protocol

The strips of antifungal A and antifungal B are crossed at a 90° angle at the position of their MICs alone. This protocol has been used to test antibiotic combinations against gram-negative and gram-positive bacteria [36], but also to test voriconazole combined with either caspofungin or amphotericin B against *Candida* spp. [37], and to test various combinations against *Candida glabrata* [38,39,40].

(ii) The fixed ratio protocol

**Figure 3:**
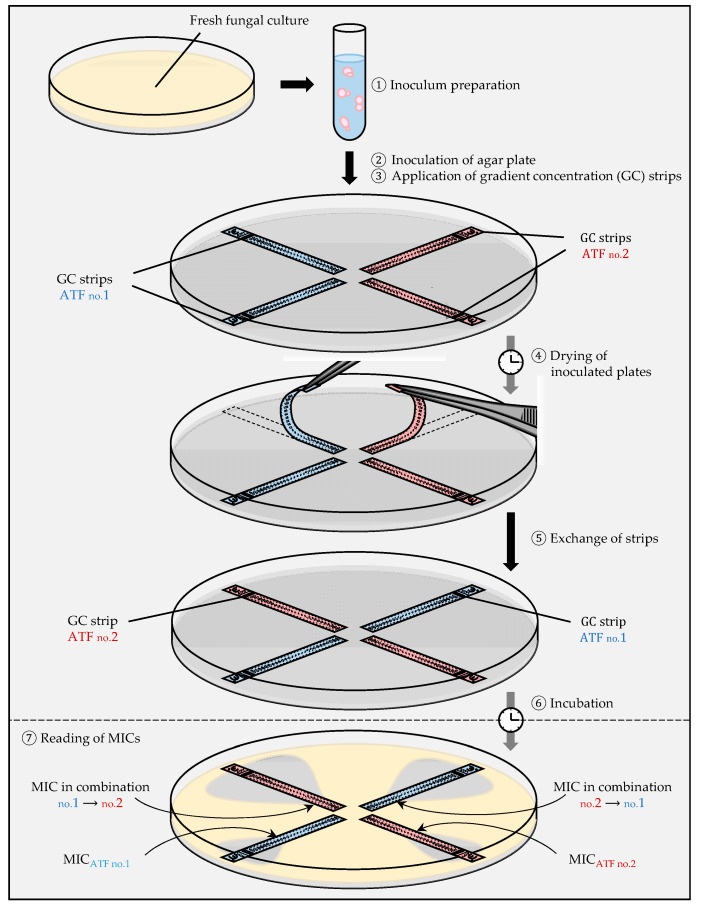
Gradient concentration strip method (Etest) for the determination of antifungal interactions: the fixed ratio protocol.

The strip of antifungal A is placed on the agar and is replaced after diffusion of the antifungal into the agar by the strip of antifungal B on exactly the same position as the first strip (Figure 3) [7,41]. This method has been used to test combinations against *C. glabrata* [38], *C. neoformans* [42], and *Aspergillus* spp. [43,44,45].

(iii) The MIC/MIC ratio protocol

The strip of antifungal A is applied onto the agar and is removed after 1 hour. After vertical transposition, the strip of antifungal B is applied on the agar surface, so the MIC of antifungal A meets the MIC of antifungal B, or a fraction of the MIC. Polymyxin B combined with fluconazole or caspofungin has been evaluated against *C. glabrata* by this method and showed synergistic interactions [46,47]. Synergy has also been found for combinations of doxycycline or tigecycline with fluconazole against *C. glabrata* [48].

The second method is used when no gradient strips are available for one of the two drugs. The MIC of antifungal A is determined by a gradient strip alone, and the MIC in combination after drug B has been incorporated in the agar at a fixed concentration [18,28,49,50,51,52,53,54,55]. A control plate with drug B alone is generally added to ensure that drug B is at a sub-inhibitory concentration. This method has been used to assess combinations of antifungals or combinations of antifungals with non-antifungal drugs against *Candida* spp. [42,49,56], *Aspergillus* spp. [18,51,54,57], and Mucorales [50].

### 2.3. Time-Kill Curves

Unlike the previous techniques which measure the inhibition of growth after a predetermined time point, time-kill curves measure the kinetics of fungicidal activity [7,41]. Fungal killing is calculated by measuring the colony forming units (CFU) at predetermined time points. The CFU are determined from tubes containing RPMI medium with the antifungals either alone or in combination. The concentrations of the antifungals are either fractions or multiples of the MICs. To interpret the results of this technique, it is necessary to compare the fungicidal activity of the combination to that obtained by the most active antifungal alone [6,7,27,41]. This method has been used to evaluate antifungal combinations or combinations of antifungals with non-antifungal drugs against *Candida* spp. [12,38,39,41,46,47,49,58,59,60,61,62,63,64,65,66,67,68,69,70,71,72,73,74,75,76,77,78,79,80,81,82,83,84,85,86], *Cryptococcus* spp. [42,59,68,70,83], and *Aspergillus* spp. [65,68,87,88,89,90,91]. Synergy or antagonism are defined by a decrease or an increase of ≥ 2 log_10_ CFU/mL of the combination compared to the most active drug [7,27]. The main advantage of this quantitative technique is the possibility to explore the fungicidal activity of combinations. The disadvantages are that technical parameters and the interpretation of the results are not standardized.

### 2.4. Analysis of Results and Interpretation

Several methods can be used to assess the combined effect of drugs that are tested in combination experiments (Figure 1).

There are several theoretical approaches to model the interaction between pharmacologically active molecules. In the field of antifungal drugs, two theories are mainly used.

The first is based on the Loewe additivity model. The model is based on the hypothesis that a drug does not interact with itself, which means the combination of a drug with itself, gives, by definition, an indifferent interaction. It is a dose-effect based strategy, meaning that concentrations that give a certain effect are compared [92]. Several methods can be applied to analyze the interactions of two drugs based on the Loewe theory, for example, intuitive graphical analysis such as the isobologram [93] or its algebraic counterpart based on the calculation of the FIC index [11]. Other approaches can also be used, such as the Greco model [94], the median-effect approach of Chou and Talabay [95], or response surface approaches [96].

The second is based on the Bliss independence model. The model is based on the hypothesis that two drugs act independently of each other. No interaction is obtained when the effect of the combination is equal to the product of the effects of the drugs alone. This approach compares the effects, instead of the concentrations, of drugs alone, or in combination. If the observed effect is better or worse than the expected indifferent interaction, the combination is defined as synergistic or antagonistic, respectively. Several methods of analysis, such as the Prichard model [97], have been developed based on the Bliss theory. Response-surface analysis can also be implemented based on the Bliss independence model [96]. Besides the Bliss independence model, other effect-based strategies can be used. These include the combination sub-thresholding, the highest single agent, and the response additivity approach [92].

#### 2.4.1. FIC Index

The fractional inhibitory concentration index, or FIC index, can be used to determine the effect of a tested combination. To determine the FIC index, the fractional inhibitory concentrations (FIC) of both drugs are added. The FIC is calculated by division of the MIC in combination and the MIC alone of the tested drug. The FIC index is calculated according to the following formula:

FIC index = FIC A + FIC B = (MIC combo1/MIC 1 alone) + (MIC combo2/MIC 2 alone).(1)

MIC 1 alone and MIC 2 alone are the MICs of antifungals 1 and 2 when tested alone, and MIC combo 1 and MIC combo 2 are the MICs of antifungals 1 and 2 in combination.

**Figure 4:**
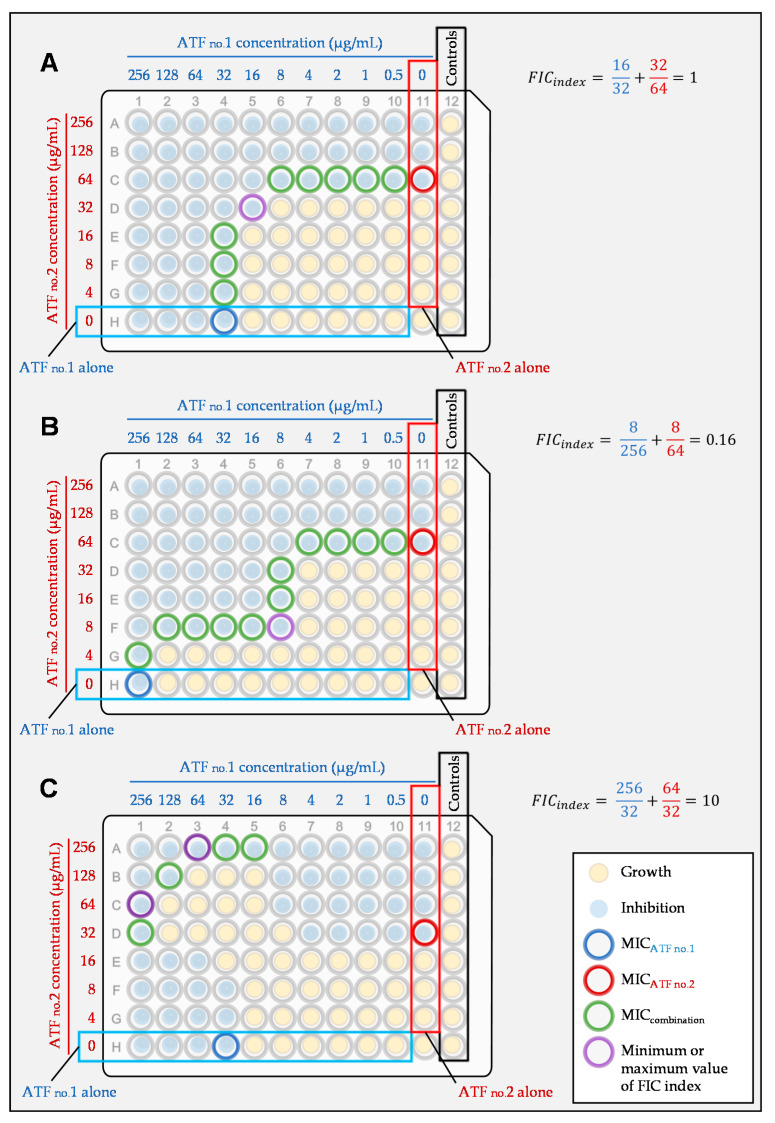
Example of a synergistic (**A**), indifferent (**B**), and antagonistic (**C**) interaction of two antifungals according to the checkerboard method and calculated by the FIC index. If there is no FIC index > 4, then the lowest FIC index is retained. If there is at least one FIC index > 4, then the highest FIC index is retained. Synergy is defined as a FIC index ≤ 0.5, indifference as a FIC index > 0.5 to 4, and antagonism as a FIC index > 4.

In theory, a FIC index = 1 represents an additivity, while a FIC index < 1 is indicative of a synergy and a FIC index > 1 of an antagonism. Nevertheless, broth microdilution techniques have an intrinsic variability of at least one log_2_ dilution. Therefore, the FIC index threshold used to analyze the results should reflect this variability. Currently, the recommendation to interpret the FIC index is as follows: interaction is synergistic when the FIC index is ≤ 0.5, indifferent if the FIC index is > 0.5 to 4, and antagonistic if the FIC index > 4 [98] (Figure 4).

With the checkerboard method, different combinations of concentrations of the antifungals are tested at the same time. It is therefore possible to calculate several FIC indices for the tested combination. The minimum FIC index is reported in absence of antagonism, and the maximum FIC index in case of antagonism. Defining the threshold of the FIC index is one of the problems of this approach, but there are others, such as the evaluation of the MIC itself. Depending on the endpoint used for MIC determination (50% or 90% of growth inhibition compared to the growth control), one can come to completely different conclusions [11,27].

#### 2.4.2. Surface Response Modeling

**Figure 5:**
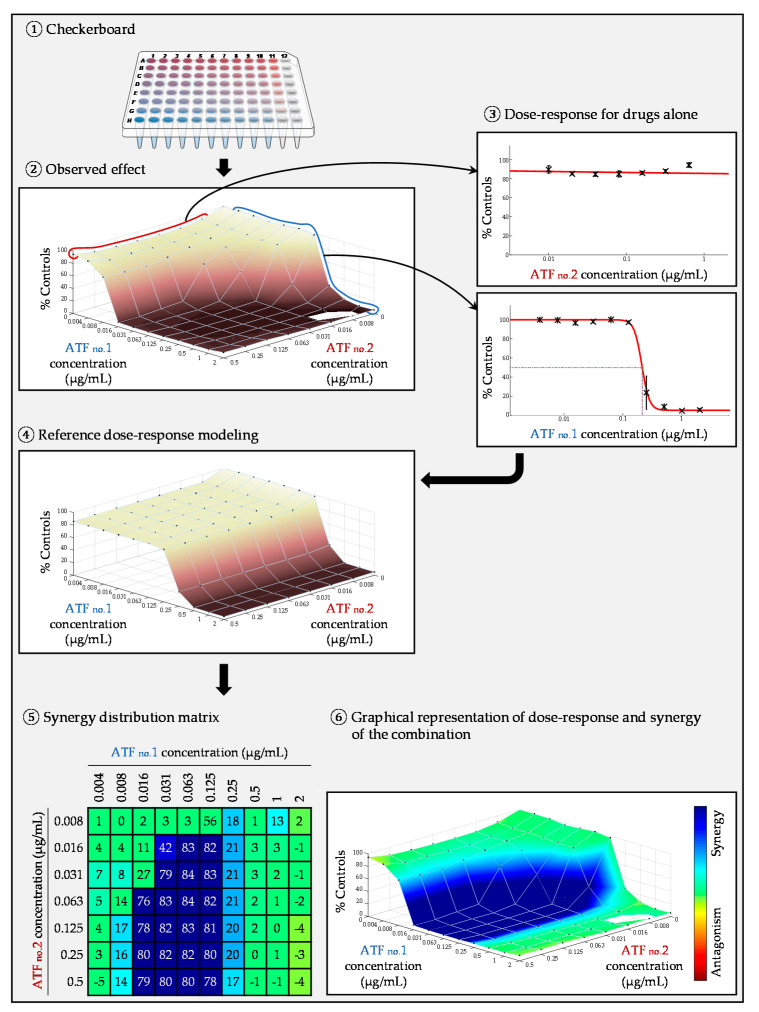
Example of a response-surface analysis for an in vitro antifungal combination experiment. Graphics were generated by the Combenefit software [96].

Response surface analysis is an alternative approach that does not require the determination of MICs. Unlike the FIC index, it is therefore independent of an inhibition endpoint. Moreover, it allows for the calculation and visualization of the combined effect of the two molecules for all tested concentrations, and not only for those corresponding to an MIC. This approach can be based on different theories (Loewe, Bliss, and other) and calculations are generally performed by dedicated software. In this approach, the inhibition curve of each antifungal agent is modeled on the basis of the growth rate obtained in each well containing the molecule alone [96]. From these dose-response curves, a theoretical growth inhibition matrix (represented by a theoretical dose-response surface) is modeled, corresponding to the inhibition rates expected in each well for the case where the interaction is purely indifferent, according to the chosen theory (Loewe, Bliss, or other model). The matrix of the experimental data (represented by an experimental dose-response surface) is then compared to the theoretical matrix. If the observed growth is weaker (stronger inhibition), synergy is concluded (Figure 5), whereas if the observed growth is stronger (weaker inhibition), antagonism is concluded. Apart of the graphical output, it is possible to generate metrics (for example the SUM-SYN-ANT metric in the Combenefit software), which can be used to quantitatively assess the drug interactions. Taking into account the intrinsic variability of the broth microdilution checkerboard technique, it is necessary to generate experimental data of the antifungals combined with themselves in order to define the threshold used for the interpretation of the metric [21,22].

## 3. In Vivo Techniques

It is important to confirm the in vitro data by in vivo data. As the incidence of most fungal infections compared to bacterial infections is lower, it is very difficult to perform clinical trials in patients, although it has been done in some instances. Combination of amphotericin B with flucytosine has been tested for the treatment of cryptococcal meningitis [99,100], or combination of voriconazole with anidulafungin for the treatment of invasive aspergillosis [101]. Therefore, animal models are essential to evaluate antifungal drug combinations in vivo.

There are no standardized techniques for testing antifungal combinations in animal models. Mammalian models (e.g., mice) are most often used. At least three groups of animals are needed to study the combination of two antifungals: one receiving the combination (A + B), one with the molecule A alone, and one with the molecule B alone. A control group of infected but non-treated animals should also be included in the experiments. The most frequently used evaluation criteria are the mortality rate and the fungal load in the target organs (determination of the number of CFU per gram of tissue by culture). To evaluate the effectiveness of the combination (mortality or number of CFUs in the organs), the group receiving the combination therapy is compared to the groups receiving monotherapy. It has to be noted that the inoculum size used to study the CFU in the organs or mortality rate is not the same. To determine the most suitable inoculum size and antifungal dosages, preliminary experiments have to be performed. To assess whether the combination is more effective than the monotherapies, the drugs alone should not give a maximum response, i.e., either a survival of all animals or a sterilization of the organs. This may therefore imply that the dosages of antifungals could be lower than those usually used in humans. Several studies of antifungal combinations in animal models of invasive candidiasis [76,102,103,104,105,106], cryptococcosis [107,108,109,110], and aspergillosis [107,111,112,113] have been realized.

Mammalian animal models have several drawbacks. Indeed, they need dedicated infrastructures, time-consuming experiments, and ethical considerations limit their use. To avoid these limitations, alternative models have been developed [114]. The *Galleria mellonella* model has been one of the most often used models in recent years [115]. The *G. mellonella* model is interesting because it is inexpensive, easy to use, and does not require a dedicated infrastructure. The larvae of *G. mellonella* are small, making them easy to handle. Additionally, the larvae can survive at 37 °C, which makes them suitable to study human fungal pathogens. This model was first used for virulence studies, but is now also used for the evaluation of antifungal combinations [116,117,118]. Larval inoculation is performed by injecting a small volume (10 µl) into a proleg on the ventral face [119]. In general, 10 to 20 larvae per group are used. Preliminary experiments to determine the lethal dose that results in 90% mortality (LD90), or the sub-lethal dose that results in 10% mortality (LD10) have to be performed according to the main endpoint (mortality or fungal load in the larvae). Most often, the main endpoint is the mortality [120,121]. *G. mellonella* has been used to test antifungal combinations against different species of yeasts and filamentous fungi. In *Candida* spp. the combination of amphotericin B and flucytosine improved the survival of infected larvae [122]. Combinations of antifungals with antibiotics have also been tested and gave similar results [123,124,125,126]. Finally, other studies have used this model to demonstrate the synergistic interaction between fluconazole and other drugs against *C. albicans* [127,128,129,130,131]. Many studies have used *G. mellonella* as a model for the evaluation of antifungal combinations against *Cryptococcus* spp. [132]. One study used the conventional antifungal agents used for the treatment of *Cryptococcus* infection (combination of amphotericin B with flucytosine) [133], another study assessed drug repurposing using the compound astemizole (antihistaminic drug) [134]. Combination therapy decreased the mortality of the larvae compared to those receiving monotherapy. This model was also used to evaluate antifungal combinations against *Aspergillus* spp. [131,135]. Combination of amphotericin B with an Hsp70 inhibitor increased survival of larvae compared to monotherapies [135]. Another study demonstrated that combination of itraconazole with EGTA (ethylene glycol tetra-acetic acid), a calcium chelator, is synergistic [131].

## 4. Conclusions

In vitro and in vivo studies of antifungal combinations are important to evaluate new therapeutic strategies in difficult-to-treat fungal infections. There are robust in vitro methods based on reference techniques, although standardization has to be improved. Advances have been made in the process of interpretation of combination results. Alternative animal models in invertebrates, which are now commonly used for testing virulence and antifungal resistance, have proven to be useful in the field of antifungal combinations. Although standardization is not fully achieved, significant results can be obtained due to the possibility of concomitantly using several techniques and several form of analysis for the interpretation of the results.

## Figures and Tables

**Table 1:** Summary of the advantages and disadvantages of the different methods used to study antifungal combinations in vitro.

Techniques	Advantages	Disadvantages
Checkerboard method	Quantitative	Discontinuous gradient of antifungal concentration
	Automated reading of results	Lack of standardization in interpretation of results
Agar diffusion assay (disks or gradient strips)	Continuous gradient of antifungal concentration	Qualitative for disks
	Possible use of commercialized systems (gradient strips)	Difficult to assess at which concentrations interaction occurs
Time-kill curves	Quantitative	Lack of standardization
	Fungicidal exploration and rate of killing	Only a few concentrations studied at the same time

## Data Availability

Not applicable.
